# Whitening Efficacy of 3% Carbamide Peroxide Gel Activated by Lactoperoxidase

**DOI:** 10.1155/2021/7143623

**Published:** 2021-05-26

**Authors:** Mehdi Khemiss, Ines Kallel, Hela Zouaghi, Mohamed Ben Khelifa, Sana Bagga

**Affiliations:** ^1^Department of Dental Medicine, Fattouma Bourguiba University Hospital, Monastir, Tunisia; ^2^Research Laboratory Functional and Aesthetic Rehabilitation of Maxillary LR12SP10, Sousse, Tunisia; ^3^Faculty of Dental Medicine, University of Monastir, Monastir, Tunisia; ^4^Research Laboratory Oral Health and Orofacial Rehabilitation LR12ES11, Monastir, Tunisia; ^5^Department of Dental Medicine, Sahloul University Hospital, Sousse, Tunisia; ^6^Research Laboratory of Biological Clinical and Dento-Facial Approach LR12ES10, Monastir, Tunisia; ^7^Department of Conservative Dentistry and Endodontics, Dental Clinic, Monastir, Tunisia

## Abstract

**Objective:**

The aim of this clinical study was to evaluate the effectiveness of a whitening toothpaste containing 3% carbamide peroxide and lactoperoxidase. *Materials and methods*. Participants were instructed to brush their teeth using an enzymatic-activated dentifrice following a particular procedure for three weeks. Color was recorded before and after treatment using a VITA Easyshade spectrophotometer. Differences in *L*^*∗*^ (tooth color lightness), *a*^*∗*^ (displacement along the red-green axis), and *b*^*∗*^ (displacement along the yellow-blue axis) were measured before and after treatment using the paired *t* and the Wilcoxon signed-rank tests. Color changes (Δ_*E*_) were calculated using the obtained measurements.

**Results:**

Thirty-four volunteers were included. Δ_*E*_ was 4.03. For the set of 348 teeth, a greater tendency towards green (lower *a*^*∗*^) and blue (lower *b*^*∗*^) was observed (*p* < 0.05). There were no significant differences in lightness after treatment. Mandibular central incisors showed a greater tendency towards green (lower *a*^*∗*^) and blue (lower *b*^*∗*^). Bleaching effectiveness was observed in both the upper and lower central incisors and in the lateral mandibular incisors.

**Conclusion:**

Based on these results, it may be concluded that brushing with an enzymatic-activated dentifrice is effective for whitening teeth.

## 1. Introduction

Tooth bleaching is one of the most requested esthetic treatments in dentistry [1]. It helps to reduce discoloration using chemical agents to oxidize organic molecules accumulated on the tooth surface [2]. Basically, there are three approaches for bleaching vital teeth: in-office or power bleaching, at-home or dentist-supervised nightguard bleaching, and bleaching with over-the-counter (OTC) products [3]. Hydrogen peroxide (HP) and its precursor, carbamide peroxide (CP), are the most widely used whitening agents [4]. First, in-office bleaching uses products delivering high concentrations of tooth whitening agents (30 to 35% HP). Peroxide can be activated by exposure to heat or light for around one hour in the dental office [5]. Remarkable whiter teeth can be seen after only one in-office treatment [5]. However, an optimum result may require several applications [6]. Secondly, at-home bleaching fundamentally involves the use of a gel applied in a fitted tray [7], containing low concentrations of whitening agents (10 to 20% CP) [3]. This technique has been used for several decades and is probably the most widely used one [8]. Finally, the popularity of OTC bleaching products has increased significantly in recent years. These products contain low concentrations of whitening agents (3 to 6% HP) and are self-applied to teeth via gum shields, strips, or paint-on product formats [9]. They are also available as prefabricated trays, whitening strips, and tubes of whitening toothpaste [9]. OTC products are considered to be the fastest-growing sector in the dental market. However, the safety of these bleaching agents may be controversial [10]. The effectiveness of most types of whitening toothpaste is based on the capacity of the abrasive particles they contain to remove external stains. They can, unfortunately, roughen the external surface of the enamel and do not change the tooth color [11]. Other types of whitening toothpaste contain peroxide. They can break down high molecular weight complex organic pigments responsible for teeth discoloration by releasing free radicals from the reaction of HP or CP. The efficiency of these kinds of toothpaste is questionable because of the low concentration of peroxide [10]. Moreover, there are concerns regarding safety issues when using these products. In fact, CP may cause gingival irritation and a change in salivary pH [12].

During the last years, a bleaching toothpaste containing 3% CP and 5% lactoperoxidase has been developed [13]. The enzyme (lactoperoxidase) has been used as an activating agent to reduce peroxide concentration in order to minimize the risk of undesirable effects [13]. The efficacy of such a low concentration remains questionable [4].

The aim of this study was to evaluate the bleaching efficacy of a bleaching toothpaste containing 3% CP and 5% lactoperoxidase and to assess its possible side-effects on the oral soft tissues. The null hypothesis tested was that there was no difference in terms of the clinical parameters between the whitening toothpaste and the nonwhitening dentifrice.

## 2. Materials and Methods

### 2.1. Study Design

This is a randomized controlled study carried out from February 2017 to April 2018 at the Department of Dental Medicine, Fattouma Bourguiba University Hospital, Monastir, Tunisia. The patients were divided into two groups: Treatment (A) and Control (B). Allocation to the two groups was randomized by a computer program. The allocation sequence was concealed in sequentially numbered envelopes (prepared by an independent researcher), and they were revealed to the main researcher at the time of the clinical examination. All the patients fulfilled the same attendance protocol.

### 2.2. Population

Participants were recruited from patients consulting the aforementioned Department of Dental Medicine from February to March 2017. Data collection was carried out during the period extending from April 2017 to February 2018. Patients were included in the study based on history-taking and clinical examination, taking into account the following inclusion criteria: age from 18 to 35 years and the presence of the 12 anterior teeth (*VITA Classical shade guide, VITA-Zahnfabrik, Bad Säckingen, Germany*). The exclusion criteria were as follows: patients with nonvital anterior teeth, tooth decay, periodontal disease, prosthetic restoration and/or resin composite restoration in the 12 anterior teeth, enamel hypoplasia, racks or fractures, dentin hypersensitivity, fluorosis, and tetracycline discoloration. In addition, patients should not have received previous teeth bleaching with dentifrice during the last six months or during the last two years in case of in-office/at-home bleaching. The two groups were pair-matched by sex, age, and socioeconomic level.

### 2.3. Sample Size

The sample size was estimated using the following formula: *n*_*A*_ = 2.*S*^2^.(*Z*_*α*/2_ − Z_1_ − _*β*_)^2^/Δ^2^ [14], where “*S*^2^” was the variance; “Δ^2^” was the minimal difference to detect; “*Z*_*α*/2_” was the normal deviate for the two-tailed alternative hypothesis at a level of significance; “*β*” was the power. Previous literature gave *S*^2^ = 3.28 [13]. Assuming a 95% confidence interval (*Z*_*α*/2_ = 1.96) [14], an 80% power (*Z*_1_−_*β*_ = −0.842) [14], and a minimal difference to detect equal to 3 [7], the total sample size was 19 for each group.

### 2.4. Whitening Dentifrice

For the treatment group, WhiteKIN bleaching treatment (*Laboratorios Kin, Barcelona, Spain*) was used. It was presented in two joined tubes, one containing a gel-based 3% CP, xylitol, and sodium fluoride, and the other containing a gel-based 5% lactoperoxidase. The content of both tubes was mixed in equal parts on the toothbrush. For the control group, toothpaste Signal kids (*Personal Care Company, Unilever Mashreq, Egypt*) was used. It was presented in a simple tube containing sodium fluoride (500 ppm fluoride) and excipients.

For the purpose of standardization, all the participants were asked to brush their teeth three times a day for three minutes during three weeks, using the product provided and following the written instructions supplied. Medium toothbrushes were used.

### 2.5. General Data

Data such as age, sex, socioeconomic status, tooth brushing frequency, cigarette consumption, and consumption of coloring drinks (coffee, tea, red wine) were collected using a French nonstandardized medical questionnaire. The questions, asked in Arabic, were with closed answers. The patients' professional level was selected to classify the socioeconomic status into low and high. Applying “*one daily tooth brushing frequency*” as a cutoff, the participants were arbitrarily classified into two subgroups: irregular (≤1) and regular (≥2) daily tooth brushing [15]. Cigarette consumption was quantified in pack-year. It was calculated by multiplying the number of packs of cigarettes smoked per day by the number of years the person had smoked.

### 2.6. Oral Data

Oral hygiene was assessed using the plaque index (PI) system of Silness and Loe, assessing the plaque thickness at the cervical margin of the tooth [16]. This system has four possible scores: 0 = no plaque; 1 = *a* film of plaque adhering to the free gingival margin which is not visible and which can be scraped from the tooth surface with a probe; 2 = moderate accumulation of soft deposits within the gingival pocket or between the tooth and the gingival margin; 3 = abundance of soft matter within the gingival pocket and/or on the tooth and the gingival margin. Twelve sites were examined using a CP 12 periodontal probe: the vestibular surface of the eight incisors and the two upper first molars and the lingual surface of the two lower first molars. A score was attributed to each site. Each participant was represented by the arithmetic mean of the 12 measurements. To assess oral hygiene evolution before and after the treatment, Δ_PI_ (PI_final value_–PI_baseline value_) was calculated.

To evaluate gingival health, the gingival index (GI) modified by Loe was used [17]. The latter evaluates the gingival condition and records the qualitative changes in order to assess the inflammatory condition of the gingiva. The criteria for the GI were as follows [17]: 0 = normal gingiva; 1 = mild inflammation, slight change in color, slight edema, and no bleeding on probing; 2 = moderate inflammation, redness, edema and glazing, and bleeding on probing; 3 = severe inflammation, marked redness and edema, ulceration, and tendency to spontaneous bleeding. The mesial, buccal, distal, and lingual sites of six teeth (16, 12, 24, 36, 32, and 44) were scored and the arithmetic mean of the scores formed the GI of the subject. To assess the difference in gingival health before and after treatment, Δ_GI_ (GI_final value_–GI_baseline value_) was calculated.

### 2.7. Salivary pH

**Figure 1:**
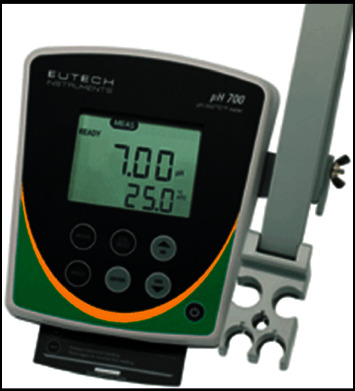
pH 700 meter.

For each subject, saliva was collected at rest. The patients were advised not to eat, drink, or perform oral hygiene for at least one hour prior to collection [18]. The participants were asked to spit the whole saliva into a 50 ml sterile Falcon® tube. Salivary pH was measured immediately after collection using a pH meter *(Eutech* pH *700 meter, Eutech Instruments, Singapore* (Figure 1). The pH meter was calibrated every day using pH_4_, pH_7,_ and pH_10_ fresh standard buffers. After analyzing the pH, the electrode tip was washed with a gentle stream of distilled water and then dipped in the double distilled water. The electrode was dipped in distilled water overnight. To assess the difference of the salivary pH before and after treatment, Δ_pH_ (pH_final value_−pH_base line_) was calculated.

### 2.8. Color Measurement

**Figure 2:**
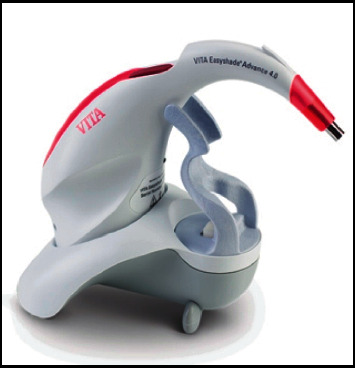
The VITA Easyshade Advance 4.0 spectrophotometer.

**Figure 3:**
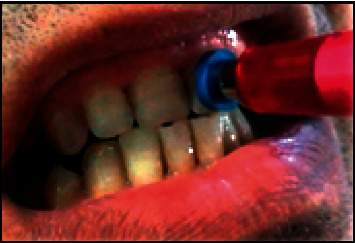
The customized positioning ring.

The tooth color parameters were performed using VITA Easyshade Advance 4.0 spectrophotometer *(VITA-Zahnfabrik, Bad Säckingen, Germany)* (Figure 2) in the same lighting conditions by the same operator (HZ) who was not involved in the randomization procedures and performed all the clinical assessments at baseline and three weeks after the bleaching treatment. Calibration of the device was performed before each measurement. To provide accurate repositioning on the middle third of the labial tooth surface, the spectrophotometer probe was surrounded by customized positioning rings having a two-millimeter thickness (Figure 3), chosen according to the coronal height. At least three measures per tooth and per appointment were noted. Repeated values were registered. Tooth color was determined according to the CIELAB spatial coordinates *L*^*∗*^, *a*^*∗*^, and *b*^*∗*^, which were provided by the apparatus. The CIELAB color scale is based on three elements: hue, chroma, and lightness (value), in which *L*^*∗*^ indicates lightness, while *a*^*∗*^ and *b*^*∗*^ represent the chromaticity coordinates [19]. Along the *a*^*∗*^ axis, positive values indicate a tendency towards red and negative values indicate a tendency towards green. Along the *b*^*∗*^ axis, negative values indicate a tendency towards blue and positive values indicate a tendency towards yellow [19].

The CIELAB Δ_E_ was then calculated in order to compare color changes using this equation [20]: Δ_*E*_ = [(Δ_*L*_)^2^ + (Δ_*a*_)^2^ + (Δ_*b*_)^2^]^1/2^, where Δ_L_ = *L*^*∗*^_final value_–*L*^*∗*^_baseline v__alue_; Δ_*a*_ = *a*^*∗*^_final value_–*a*^*∗*^_baseline value_; and Δ_*b*_ = *b*^*∗*^_final value_–*b*^*∗*^_baseline value_.

The tooth shades according to both international shade standards (VITA Classical and VITA system 3D-MASTER) were displayed and recorded by the apparatus. The VITA Shade Guide has 16 tooth tabs with different color shades. Shades were arranged from highest (B1) to lowest (C4) value as follows: B1, A1, B2, D2, A2, C1, C2, D4, A3, D3, B3, A3.5, B4, C3, A4, and C4 [7].

### 2.9. Statistical Analysis

All the mathematical computations and the statistical procedures were performed using SPSS software (Social Package Statistical Sciences, version 18, USA). Significance was set at 0.05 level. To assess the normal distribution of quantitative variables, the Kolmogorov–Smirnov normality test was used. If variables had a normal distribution, they were expressed as mean ± standard deviation (SD) and then compared by means of a Student's *t*-test for paired samples. If not, they were expressed as median [First Quartile-Third Quartile] and then compared using the Wilcoxon test. Qualitative variables were expressed as frequency and percentages. The Chi^2^ of McNemar test was used to compare the two groups' qualitative data.

### 2.10. Ethical Consideration

The study was conducted in accordance with the Declaration of Helsinki and its ethical principles for medical research involving human subjects.

The committee of person of Monastir Tunisia, which is responsible for ensuring the clinical trials in Tunisia, was contacted. Its approval was not obtained since this whitening dentifrice is still authorized in Tunisia. Therefore, the study was approved by the local ethics committee (approval number: 07052021).

All the participants were enrolled voluntarily without any direct benefits. They were individually informed about the purpose of the study. After providing the patients with a detailed explanation of the study characteristics, informed consent was obtained. The subjects diagnosed with any oral pathology were given treatment or were referred to the right specialist.

## 3. Results

**Figure 4:**
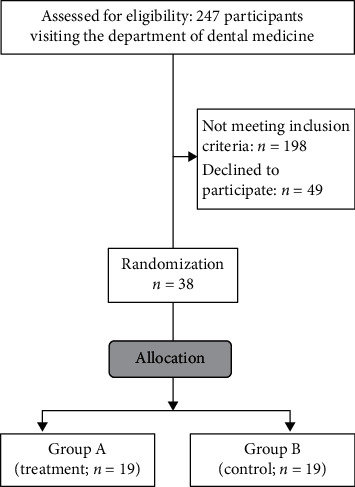
Flowchart of participants included in the study.

Among the 247 patients assessed for eligibility, noninclusion criteria were found in 198 (Figure 4). Forty-nine did not accept to take part in the study. Therefore, the final sample included 38 participants, which were randomized into two groups (19 in the treatment group, 19 in the control group).

The sociodemographic information and the hygienic and dietary habits of the two groups are summarized in Table 1. No significant differences were found between the groups regarding tooth brushing, tobacco use, coloring drinks, and prior bleaching treatment. The two groups were pair-matched by age, gender, and socioeconomic level.

Table 2 displays the oral data (PI and GI) and the pH values of the two groups. The two groups had similar PI, Δ_PI_, Δ_GI_, pH, and Δ_pH_. A statistically significant (*p* = 0.01) difference between the two groups' GI (baseline values) was observed.

Tables 3 and 4 display the CIELAB spatial coordinates *L*^*∗*^, *a*^*∗*^, *b*^*∗*^, and Δ_E_, respectively, for the whole sample and for each anterior tooth. No significant difference was found between the two groups' CIELAB spatial coordinates values, except for the Δ_E_ value of the right maxillary lateral incisor, which was significantly higher in the treatment group compared to the control group (respectively, 6.46 ± 4.67 vs. 4.27 ± 2.42, *p* = 0.04).

Table 5 displays Δ_E_ values for each tooth shade. A significant difference between the two groups was noted only in shade A3.5 (*p* < 0.01).

## 4. Discussion

The main result of the present study revealed that whitening toothpaste containing 3% CP and 5% lactoperoxidase tended to offer clinically satisfactory results in terms of tooth color modifications. However, no significant differences were found in comparison to the control group.

### 4.1. Discussion of the Results

The baseline GIs were significantly higher in the treatment group compared to those in the control group (*p* = 0.01). Changes in the GIs from baseline did not differ significantly between the two groups. These results were in accordance with a study evaluating the possible long-term adverse effects of peroxide-containing agents on the oral soft tissues [21]. The aforementioned study showed no statistically significant differences in the degree of oral mucosal irritation between patients using 10% CP whitening gel and those using a placebo gel during a two-week treatment period [21]. The oral effects were evaluated only over a three-week period. This may be a limitation in our study since subsequent complications may exist. However, no delayed adverse effects on the oral soft tissues were noted when using low-level doses of CP [22]. This may be explained by the addition of lactoperoxidase, which provides protection for the oral soft tissues when added to a toothpaste [4].

The baseline pH was similar in both groups. The mean pH in both groups showed an increase from baseline when compared to the final value, but no statistically significant difference was noted between the two groups. This stability of pH values is very important because it may have an influence on the properties of tooth enamel subjected to whitening [1]. In fact, low pH whitening toothpaste is responsible for in vitro morphological changes in the enamel [3].

The treatment group showed a ∆_E_ mean value of 5.34 units. In the control group, we also recorded improvements in tooth color based on the ∆_E_ mean value which was 4.32 units. These variations may have been due to the cleaning properties of the ordinary toothpaste despite its low abrasiveness and the optimal oral hygiene of patients who were committed to brushing their teeth three times a day [4, 23]. There was no significant difference in displacement towards green (Δ_a_) and towards blue (Δ_b_) between the two groups. As for lightness, an increase was observed in the treatment group. However, a decrease was noted in the control group but with no significant difference. These results were in accordance with the findings of another clinical trial, evaluating the same toothpaste used in our study [4]. The studies using different OTC products containing peroxide at low concentrations offer contradictory results [9]. A three-week clinical trial showed no significant difference in color improvement for the 18% CP paint-on gel compared to normal brushing with a dual-phase whitening dentifrice [24]. In contrast, a clinical study, investigating the whitening effectiveness of toothpaste delivering 1% HP versus nonwhitening toothpaste, recorded significant improvement in tooth shade in the active treatment groups after four weeks of use [25], although the 3% CP used in our study delivered similar HP concentration of 1.08%. A previous clinical study using the same product revealed improvement in the color of the treated teeth when applied for three weeks, with a similarly significant increase in brightness at the end of treatment [13]. To allow more shade change, the application period could be extended since there is a significant impact of both duration and concentration on color change [9]. However, the study about the same toothpaste did not find a significant difference in color change in the control group after three and twelve weeks of application [4].

The results showed a significantly higher Δ_*E*_ value for the right maxillary lateral incisor in the treatment group. This difference in bleaching efficacy between the right and left sides may be due to the difference in the brushing efficacy between the two sides. In fact, right-handed people would brush harder and more efficiently their left teeth. Consequently, the left teeth were initially more bleached than the right ones, which were more likely to respond to bleaching treatment. Besides, the bleaching procedure used is a patient-dependent technique where the subject's commitment to the provided protocol and instructions plays a major role in the bleaching process [11]. This may be a limitation of the present study since the brushing frequency and the amount of toothpaste used were not regularly controlled. However, no significant difference between the left and the right was detected for both the central incisors and canines, although they were both brushed simultaneously with the same efficacy. This difference may be explained by the initial values of color coordinates of the maxillary lateral incisor (Table 4). In fact, the right incisors in the treatment group were initially more chromatic (reddish and yellowish) than the left ones. This result may also suggest a higher bleaching efficacy of the tested toothpaste on the darker shades. To investigate this idea, the Δ_*E*_ values for each tooth shade (according to VITA Classical shade guide) were compared. The only significant difference between the two groups (treatment and control) was for shade A3.5. This finding pleads in favor of the above-cited suggestion. In fact, A3.5 was the second darkest shade in our sample after A4 [26]. The absence of significant difference for shade A4 may be due to the small number (*n* = 4) of teeth having this shade in our sample (Table 5). No studies evaluating color change depending on teeth shade were found. However, many researchers defined a minimal tooth shade as an inclusion criterion without reaching an agreement on this issue [4, 13, 26]. In fact, while Forner et al. [13] included participants having B2 or darker tooth color in their clinical study, the subjects included in the study conducted by Liena et al. [4] had A3 or higher tooth shade. However, Ontiveros et al. [26] excluded participants having a shade lighter than A2. Further studies might be helpful to assess the bleaching efficacy according to the initial tooth shade and to corroborate our findings to identify A3.5 and darker shades as better responding to bleaching with the 3% CP and 5% lactoperoxidase.

### 4.2. Discussion of the Methodology

This was a controlled randomized clinical study. This study design helps to establish or verify certain therapeutic data (efficacy and safety) of a new drug or a new way in which a known treatment is used. However, this study was blinded only to the evaluator. This was considered as a limitation since double blinding provides the ultimate proof of effectiveness. In this study, double blinding was not possible for logistic and financial reasons. Therefore, the motivation of the controls and their commitment to the treatment protocol might have been affected [27].

Thirty-eight subjects were recruited (19 for each group). The sample size was calculated according to a predictive equation before the beginning of the study [14]. Calculation of the sample size is a statistically central point since determining the sample finest size guarantees enough power to distinguish statistical significance [14].

In both groups, age, gender, and socioeconomic level were matched. Hygienic and dietary habits showed no significant differences between the two groups. Therefore, the bleaching efficacy would not be affected and the two groups were comparable. Moreover, all the subjects were required to follow the same instructions to guarantee optimal conditions for tooth bleaching. Only participants aged from 18 to 35 years were included in order to standardize specimens in terms of teeth development and maturation [27]. In fact, a significant relationship between the subjects' age and the magnitude of whitening response was reported [28]. Older subjects with less yellow initial tooth color showed the smallest postbleaching mean color change, while younger subjects with more yellow initial tooth color manifested the greatest postbleaching mean color change [8].

Cervical tooth decay, nonadapted prosthetic restoration, enamel hypoplasia, cracks or fractures, resin composite restoration, tooth fluorosis, and tetracycline discoloration in the 12 front teeth were applied as noninclusion criteria. In fact, the enamel can be colored with saliva and food. Its surface condition and permeability at the cracks and fissures play an important role in tooth staining. In addition, the final result of tooth bleaching can be significantly affected by the type of intrinsic stain. Mild-to-moderate tetracycline staining requires bleaching regimes extending from two to six months. On the other hand, restorations in the anterior sector may show noticeable color change after the bleaching treatment [29]. Since CP leads to tooth sensitivity and gingival inflammation [12], these two conditions were also applied as noninclusion criteria. Finally, the subjects who had undergone prior bleaching treatment with dentifrice (<6 months earlier) or in-office/at-home bleaching (<2 years before) were not included to avoid a possible accumulative effect.

For the treatment group, WhiteKIN bleaching treatment (toothpaste + gel) (*Laboratorios Kin, Barcelona, Spain*) was used. Unlike most types of whitening toothpaste, it has low abrasiveness, indicated by its relative dentin abrasivity (RDA) index of 56. This product contains 3% CP and 5% lactoperoxidase in order to reduce peroxide concentration, thus minimizing the risk of undesirable effects [13]. In fact, in recent years, many researchers in dental bleaching have tried to define the activation mechanisms capable of affording optimum free radical action with the lowest HP concentration possible. Many activation mechanisms have been developed, such as the use of physical agents, including different light sources, chemicals, and enzymes [4]. Lactoperoxidase is one of the enzymes that can be utilized in enzymatic bleaching [4, 13]. It acts by catalyzing HP decomposition and transforming it into a harmless substance [30]. Moreover, its stability allows its incorporation in gel formulations [31]. For the control group, Signal Kids Toothpaste (*Personal Care Company, Unilever Mashreq, Egypt*) was chosen based on its relative RDA index, which was the closest to that of WhiteKIN on the market. In fact, every toothpaste requires a certain level of abrasivity to remove plaque, debris, and stains from accessible tooth surfaces without damaging the tissues [32]. Each toothpaste has an index of abrasiveness determined by the characteristics of the abrasive particles. Since most types of toothpaste claiming to have whitening properties present a higher abrasiveness index [33], it was important to use two kinds of toothpaste with similar RDA indexes.

A spectrophotometer was used to determine the tooth shade. Color measurement devices have been used in bleaching studies to document shade changes. Spectrophotometers, colorimeters, and imaging systems are known to be useful and relevant tools for tooth color measurement and analysis [34]. These devices have benefits and limitations. In fact, the clinician has to consider the appropriate technology for their expectations and needs. Spectrophotometers are among the most accurate, useful, and flexible instruments for color matching in general and for color matching in dentistry [35]. Spectrophotometers have shown to be more precise than observations by the human eye or conventional techniques [36].

Color variations were evaluated based on ∆_*E*_. There is a consensus that the determination of Δ_*E*_ is appropriate to define acceptability tolerances and perceptibility tolerances since this clinical parameter has a visual significance [4]. However, different values have been used and no consensus has been established [37]. In this context, it is accepted that variations in ∆_*E*_ of ≥2.7 units indicate color changes, while variations of ≥3.3 units are indicative of clinically obvious changes in tooth color [38]. The Δ_*E*_ retained in the present study was 3 [7].

## 5. Conclusion

Brushing with bleaching toothpaste containing 3% CP and 5% lactoperoxidase seems to be effective for tooth whitening after a three-week exposure time. However, this enzymatic-activated dentifrice did not change the tooth shade when compared to a placebo and nonwhitening dentifrices. Future studies are needed to assess color reversion of the results after a long period of time.

## Figures and Tables

**Table 1:** Characteristics of the treatment group (*n* = 19) and the control group (*n* = 19).

		Treatment	Control	*p*
Data in mean ± standard deviation				
Age		26.2 ± 4.2	26.2 ± 4.2	—
Data in number (percentage)				
Gender	Male	6 (32)	6 (32)	—
	Female	13 (68)	13 (68)	

Socioeconomic level	Low	0 (0)	0 (0)	—
	High	19 (100)	19 (100)	

Tooth brushing	<2	6 (32)	2 (11)	0.23
	≥2	13 (68)	17 (89)	

Tobacco use	Yes	3 (16)	0 (0)	0.23
	No	16 (84)	19 (100)	

Coloring drinks	Yes	14 (74)	18 (95)	0.18
	No	5 (26)	1 (5)	

Bleaching treatment	Yes	3 (16)	1 (5)	0.60
	No	16 (84)	18 (95)	

**Table 2:** Plaque index, gingival index, and potential hydrogen of the treatment group (*n* = 19) and the control group (*n* = 19).

	Treatment	Control	*p*
Data in mean ± standard deviation			
PI_baseline value_	0.56 ± 0.45	0.37 ± 0.34	0.16
Δ_PI_	−0.24 ± 0.47	−0.25 ± 0.25	0.14
GI_baseline value_	0.45 ± 0.31	0.17 ± 0.19	0.01
Δ_GI_	−0.25 ± 0.30	−0.13 ± 0.13	0.13
pH_baseline value_	6.83 ± 0.35	6.87 ± 0.4	0.77
Δ_pH_	0.04 ± 0.51	0.18 ± 0.3	0.40

^*∗*^ *p* < 0.05 (*t*-test): treatment group vs. controls.

**Table 3:** *L* ^*∗*^, *a*^*∗*^, *b*^*∗*^, and ΔE values for the treatment group (*n* = 19) and the control group (*n* = 19).

	Treatment	Control	*p*
Data in mean ± standard deviation			
Δ_L_	0.16 ± 3.64	−1.62 ± 3.34	0.20
Δ_a_	−0.90 ± 0.40	−0.12 ± 0.31	0.81
Δ_b_	0.02 ± 2.22	−0.43 ± 0.95	0.48
Δ_E_	5.34 ± 2.31	4.32 ± 1.56	0.16

**Table 4:** *L* ^*∗*^, *a*^*∗*^, *b*^*∗*^, and ΔE values for each tooth of the treatment group (*n* = 19) and the control group (*n* = 19).

		Treatment	Control	*p*
Data in mean ± standard deviation				
11	Δ_*L*_	−0.88 ± 4.94	−1.47 ± 4.43	0.74
	Δ_*a*_	−0.26 ± 0.89	−0.03 ± 0.38	0.23
	Δ_*b*_	−1.02 ± 3.20	0.69 ± 2.80	0.09
	Δ_*E*_	4.59 ± 3.91	4.61 ± 2.81	0.98

12	Δ_*L*_	−0.89 ± 6.15	−1.84 ± 4.17	0.62
	Δ_*a*_	0.01 ± 0.88	−0.02 ± 0.54	0.90
	Δ_*b*_	0.45 ± 5.12	−0.12 ± 1.97	0.67
	Δ_*E*_	6.46 ± 4.67	4.27 ± 2.42	0.04^*∗*^

13	Δ_*L*_	0.09 ± 5.31	−2.67 ± 3.61	0.11
	Δ_*a*_^*a*^	−0.1[−0.4; 0.1]	0.1[−0.2; 0.3]	0.37
	Δ_*b*_	0.38 ± 4.00	−0.74 ± 2.46	0.35
	Δ_*E*_	5.24 ± 4.04	4.59 ± 2.27	0.58

21	Δ_*L*_	−0.89 ± 7.29	−1.39 ± 3.36	0.82
	Δ_*a*_	0.10 ± 1.04	0.18 ± 0.47	0.71
	Δ_*b*_	−0.38 ± 3.22	1.12 ± 3.27	0.18
	Δ_*E*_^*a*^	5.1[2.9; 8.4]	4.8[2.8; 5.5]	0.24

22	Δ_*L*_	0.53 ± 5.04	−1.26 ± 3.27	0.28
	Δ_*a*_	0.12 ± 4.47	0.15 ± 0.57	0.86
	Δ_*b*_	0.48 ± 3.63	0.74 ± 2.61	0.80
	Δ_*E*_	5.06 ± 3.52	3.65 ± 2.45	0.22

23	Δ_*L*_	−0.60 ± 6.25	−1.71 ± 2.77	0.50
	Δ_*a*_	0.11 ± 0.63	−0.01 ± 0.56	0.58
	Δ_*b*_	0.18 ± 2.56	−0.47 ± 1.61	0.30
	Δ_*E*_	5.44 ± 3.90	3.24 ± 1.67	0.06

31	Δ_*L*_	0.50 ± 4.58	−2.55 ± 4.45	0.10
	Δ_*a*_	−0.44 ± 0.82	−0.26 ± 0.52	0.45
	Δ_*b*_	−0.29 ± 3.62	−0.97 ± 2.16	0.53
	Δ_*E*_	5.10 ± 2.81	4.71 ± 3.05	0.65

32	Δ_*L*_	1.29 ± 5.27	−1.41 ± 3.95	0.13
	Δ_*a*_	−0.21 ± 0.97	−0.26 ± 0.85	0.88
	Δ_*b*_	0.57 ± 3.72	−0.52 ± 1.60	0.27
	Δ_*E*_	5.71 ± 3.21	4.01 ± 2.10	0.11

33	Δ_*L*_	0.91 ± 6.30	−0.52 ± 3.89	0.46
	Δ_*a*_	0.22 ± 0.76	0.10 ± 0.52	0.63
	Δ_*b*_	0.98 ± 5.04	−0.30 ± 2.00	0.35
	Δ_*E*_	6.38 ± 4.97	3.78 ± 2.18	0.06

41	Δ_*L*_	1.25 ± 4.59	−2.08 ± 4.81	0.07
	Δ_*a*_	−0.29 ± 0.83	0.01 ± 0.55	0.24
	Δ_*b*_	−0.05 ± 2.60	0.07 ± 1.91	0.89
	Δ_*E*_	4.79 ± 2.47	4.65 ± 2.96	0.89

42	Δ_*L*_	0.61 ± 4.67	−1.01 ± 5.29	0.41
	Δ_*a*_	−0.45 ± 0.73	−0.44 ± 0.7	0.95
	Δ_*b*_	−0.95 ± 2.33	−0.50 ± 2.02	0.51
	Δ_*E*_	4.64 ± 2.59	4.83 ± 3.07	0.83

43	Δ_*L*_	0.03 ± 4.04	−1.52 ± 5.87	0.34
	Δ_*a*_	−0.13 ± 0.50	−0.15 ± 0.62	0.89
	Δ_*b*_^*a*^	0.1[−1.9; 1.4]	−0.1[−1.5; 1.2]	0.71
	Δ_*E*_	4.17 ± 1.89	5.06 ± 4.07	0.38

^a^Data are median (first quartile-third quartile).^*∗*^*p* < 0.05 (*t*-test): treatment group vs. controls.

**Table 5:** ΔE values for each shade of the treatment group and the control group (*n* = 19).

	Treatment	Control	*p*
Data in median (first quartile-third quartile)			
A1	4.39 [2.36–5.76] *n* = 52	3.60 [2.03–5.48] *n* = 60	0.209
A2	3.97 [2.89–5.09] *n* = 24	3.18 [1.77–5.17] *n* = 47	0.206
A3	4.69 [2.43–8.10] *n* = 21	5.15 [4.27–7.28] *n* = 13	0.425
A3.5	4.08 [3.33–7.06] *n* = 19	2.36 [1.74–4.08] *n* = 16	0.004
A4	7.28 [2.98–10.57] *n* = 4	8.08 [4.75–11.97] *n* = 7	0.705
B1	5.12 [2.31–12.99] *n* = 20	4.82 [3.70–9.23] *n* = 11	0.741
B2	3.95 [2.28–6.53] *n* = 37	3.31 [1.80–6.44] *n* = 28	0.529
B3	4.30 [2.86–7.86] *n* = 36	3.71 [2.57–5.96] *n* = 40	0.206

^*∗*^ *p* < 0.05 (test of Mann–Whitney): treatment vs. controls.

## Data Availability

All data are available within the manuscript.
